# Metabolic dynamics restricted by conserved carriers: Jamming and feedback

**DOI:** 10.1371/journal.pcbi.1005847

**Published:** 2017-11-07

**Authors:** Tetsuhiro S. Hatakeyama, Chikara Furusawa

**Affiliations:** 1 Department of Basic Science, Graduate School of Arts and Sciences, The University of Tokyo, Meguro-ku, Tokyo, Japan; 2 Quantitative Biology Center (QBiC), RIKEN, Suita, Osaka, Japan; 3 Universal Biology Institute, The University of Tokyo, Bunkyo-ku, Tokyo, Japan; University of Michigan, UNITED STATES

## Abstract

To uncover the processes and mechanisms of cellular physiology, it first necessary to gain an understanding of the underlying metabolic dynamics. Recent studies using a constraint-based approach succeeded in predicting the steady states of cellular metabolic systems by utilizing conserved quantities in the metabolic networks such as carriers such as ATP/ADP as an energy carrier or NADH/NAD^+^ as a hydrogen carrier. Although such conservation quantities restrict not only the steady state but also the dynamics themselves, the latter aspect has not yet been completely understood. Here, to study the dynamics of metabolic systems, we propose adopting a carrier cycling cascade (CCC), which includes the dynamics of both substrates and carriers, a commonly observed motif in metabolic systems such as the glycolytic and fermentation pathways. We demonstrate that the conservation laws lead to the jamming of the flux and feedback. The CCC can show slow relaxation, with a longer timescale than that of elementary reactions, and is accompanied by both robustness against small environmental fluctuations and responsiveness against large environmental changes. Moreover, the CCC demonstrates robustness against internal fluctuations due to the feedback based on the moiety conservation. We identified the key parameters underlying the robustness of this model against external and internal fluctuations and estimated it in several metabolic systems.

## Author summary

Although a metabolic shift is essential for the adaptation of cells or organisms to environmental changes, the transient behaviors of metabolic systems are poorly understood. When describing the time development of metabolic systems, there are several conserved quantities to consider due to balances of metabolic reactions, e.g., the cycling of coenzymes. Such conserved quantities limit the possible changes in the metabolic state and can generate non-trivial dynamical behaviors. We here propose a minimal motif of metabolic reactions that includes coenzyme recycling to investigate the effect of conserved quantities on metabolic dynamics. We demonstrate that the dynamics with this motif intrinsically show slow relaxation to the steady state after environmental changes. Moreover, this motif can maintain robustness against external and internal fluctuations owing to the conservation of coenzymes. Overall, these results suggest that the complex metabolic dynamics generated by coenzyme recycling are beneficial to organisms.

## Introduction

In recent decades, mathematical modeling of metabolic systems has been intensively explored in the field of systems biology [1–6]. Several studies have demonstrated that metabolic systems can be quantitatively predicted using mathematical models [7–10]. A particularly important feature for the mathematical modeling of a metabolic system is that some conserved quantities characterize the system. The total number of atoms remains unchanged before and after the reactions, allowing for mass conservation. Furthermore, several coenzymes act as the carriers of molecules and energy through various reactions, e.g., ATP/ADP as an energy carrier and NADH/NAD^+^ as a hydrogen carrier [11, 12]. The total concentrations of the carriers represent the conserved quantities in the steady state, which is referred to as moiety conservation [11], by which various coenzyme-related reactions have to be balanced. These constraints, i.e., the law of mass conservation and moiety conservation, restrict the solution space and facilitate the analysis of complicated metabolic networks. One of the most successful approaches used for the modeling of metabolic systems to date is the constraint-based analysis of metabolic fluxes based on the stoichiometry that describes conservation laws in metabolic fluxes [2, 5]. In this approach, the metabolic fluxes are assumed to be in a steady state, meaning that, for any metabolite pool, the fluxes governing its synthesis and degradation are balanced. Owing to its predictive power and advantage of not requiring detailed information on the kinetic parameters, a steady state-based metabolic modeling approach has been applied in various studies analyzing the characteristics of metabolic systems.

However, in contrast to the steady-state solution, the characteristics of the dynamic behavior of metabolic systems have not been elucidated. In a fluctuating environment, a metabolic system is not in a steady state, and thus its dynamic behavior should be analyzed over time. In a biological system, the producing and decomposition reactions of such carriers are also faster than cellular growth. Since the conservation laws are considered to be maintained for a longer time than several cell cycles, the law of mass conservation and moiety conservation restrict both the steady state and the transient dynamics. Thus, the constraints of metabolic networks should be taken into account when studying metabolic dynamics. In particular, the moiety conserved coenzymes should have particularly strong effects on the various pathways in analyzed metabolic systems due to their recycling [11]. For example, in the glycolytic pathway, ATP is transformed into ADP by phosphofructokinase, while ADP is recycled to ATP in downstream reactions. During the anaerobic growth of microorganisms, NAD^+^ is transformed into NADH by glyceraldehyde-3-phosphate dehydrogenase in upper glycolysis, while NADH is recycled to NAD^+^ through the fermentation pathways [1]. These reactions involving carriers must be balanced not only in the steady state but also in the dynamic states, and yet the effect of such balance remains unknown.

Several previous studies have analyzed the characteristics of metabolic pathways with moiety conservation. Reich and Sel’kov [11] derived simple dynamical system models of metabolic system, and pointed out that recycling of the moiety conservations represents the skeleton of energy metabolism. Such a moiety-conserved cycle was formulated using mass-action kinetics and was analyzed with dynamical systems theory. They found that the cycle of moiety conservation works as a positive feedback to produce the high-energy carrier autocatalytically if its concentration is low, whereas a higher concentration of the high-energy carrier limits its own production. Following these pioneering works, the behavior of the moiety-conserved metabolic cycle has been extensively studied [13, 14]. Although the majority of these studies focused only on the analysis of steady states, some researchers addressed the dynamic behavior of the moiety-conserved cycle after environmental changes [15, 16]. These studies demonstrated transient switching behavior based on a simple model with mass-action kinetics without considering complex formation among enzymes, substrates, and cofactors.

Recently, some theoretical studies demonstrated that complex formation in a chemical reaction system can cause transient abnormal behavior, which could not be obtained using simple mass-action kinetics [17]. This finding suggested that the complex dynamic behavior of metabolic systems, especially those including moiety conservation, can also be captured by considering complex formation. Following this idea, in the present study, we focused on metabolic reaction pathways with carrier recycling (also known as a “turbo design” [18]). To investigate the dynamic behavior of nonlinear metabolic systems with the law of mass conservation and moiety conservation, we here explore a simple motif designated as a carrier cycling cascade (CCC), which simultaneously considers the dynamics of substrates and coenzymes. We formulate the CCC with complex formation to analyze the dynamics of both substrates and carriers simultaneously. The CCC shows slow relaxation into the steady state against external perturbations around a critical point where the relaxation speed follows the power law. We analyze the origin of the slow relaxation using dynamical systems theory. Additionally, the CCC maintains robustness of a metabolic state with intrinsic noise. We demonstrate that the negative feedback through the conserved carrier provides such robustness, i.e., the moiety-conserved cycle can reduce fluctuations in the concentrations of metabolites. We further discuss the relationship between microscopic feedback and macroscopic dynamics, and estimate the effect of this feedback using experimentally determined parameters.

## Results

### Modeling of the carrier cycling cascade

**Fig 1:**
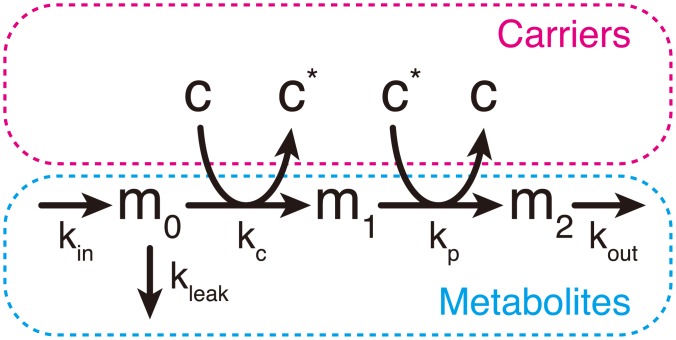
A simple model of the carrier cycling cascade (CCC). The model comprises the metabolites and carriers. Although the total concentration of metabolites can change with time, that of the carriers remains constant.

The steady-state characteristics in a moiety-conserved cycle were intensively studied by Reich and Sel’kov [11]. However, most of these proposed models are too complicated to effectively extract the essence of the dynamic features. The metabolite flow is usually branched, and the same coenzyme can be utilized at multiple steps. Hence, to investigate the effects of a cycling carrier on the dynamics of metabolic systems, we here focus on the simplest cascade, the CCC, which models ATP-ADP cycling in the glycolysis pathway and NADH-NAD^+^ cycling in the fermentation pathway (Fig 1). Here, we refer to the high-energy and low-energy carriers as the active and inactive carriers, respectively. The CCC consists only of active carrier-consuming and -producing steps.

Our model is similar to the simplest skeleton proposed by Reich and Sel’kov [11]. In contrast to the previous study, which considered the mass-action dynamics without complex formation, we here include the complex-formation process. We assume that the enzyme, substrate, and coenzyme form the complex, while the enzyme is saturated under the ordinal metabolic condition [19, 20] and does not appear in the equation explicitly.

Original ordinary differential equations (ODEs) are given as seven mass-action kinetic equations (see section 1 in S1 Text), which can be reduced to five ODEs by eliminating the association and dissociation reactions between coenzymes and substrates adiabatically.

$$\begin{array}{ccc} \frac{d[m_{0}]}{dt} & = & k_{\text{in}}-k_{c}\frac{\left[m_{0}\right]\left[c\right]}{K_{0}+\left[c\right]}-k_{\text{leak}}\left[m_{0}\right], \end{array}$$ (1a)

$$\begin{array}{ccc} \frac{d[m_{1}]}{dt} & = & k_{c}\frac{\left[m_{0}\right]\left[c\right]}{K_{0}+\left[c\right]}-k_{p}\frac{\left[m_{1}\right]\left[c^{*}\right]}{K_{1}+\left[c^{*}\right]}, \end{array}$$ (1b)

$$\begin{array}{ccc} \frac{d[m_{2}]}{dt} & = & k_{p}\frac{\left[m_{1}\right]\left[c^{*}\right]}{K_{1}+\left[c^{*}\right]}-k_{\text{out}}\left[m_{2}\right], \end{array}$$ (1c)

$$\begin{array}{ccc} \frac{d{\left[c\right]}_{t}}{dt} & = & -k_{c}\frac{\left[m_{0}\right]\left[c\right]}{K_{0}+\left[c\right]}+k_{p}\frac{\left[m_{1}\right]\left[c^{*}\right]}{K_{1}+\left[c^{*}\right]}, \end{array}$$ (1d)

$$\begin{array}{ccc} \frac{d{\left[c^{*}\right]}_{t}}{dt} & = & k_{c}\frac{\left[m_{0}\right]\left[c\right]}{K_{0}+\left[c\right]}-k_{p}\frac{\left[m_{1}\right]\left[c^{*}\right]}{K_{1}+\left[c^{*}\right]}, \end{array}$$ (1e)

$$\begin{array}{ccc} {\left[c\right]}_{t} & = & \left[c\right]+\frac{\left[m_{0}\right]\left[c\right]}{K_{0}+\left[c\right]}, \end{array}$$ (1f)

$$\begin{array}{ccc} {\left[c^{*}\right]}_{t} & = & \left[c^{*}\right]+\frac{\left[m_{1}\right]\left[c^{*}\right]}{K_{1}+\left[c^{*}\right]}, \end{array}$$ (1g)

where *m*_*i*_ is the *i*-th metabolite, *c* and c* are active and inactive carriers, respectively, and [*x*] denotes the concentration of *x*. *m*_0_ is supplied and diluted with rates *k*_in_ and *k*_leak_, respectively, and *m*_2_ is diluted with rate *k*_out_. *c* and *m*_0_ make complex *cm*_0_, and c* and *m*_1_ make complex c* *m*_1_. The active carrier is consumed with rate *k*_c_ when *m*_1_ is transformed from *m*_0_, and is produced with rate *k*_p_ when *m*_2_ is transformed from *m*_1_. [*c*]_t_ and [c*]_t_ represent the total concentration of active and inactive carriers as [*c*]_t_ = [*c*] + [*cm*_0_] and [c*]_t_ = [c*] + [c*m_1_], respectively. *K*_0_ and *K*_1_ represent the dissociation constants between *c* and *m*_0_ and between c* and *m*_1_, respectively.

Here, we considered the condition in which the total concentrations of the carriers are conserved. There are multiple reactions between the active carrier-producing and -consuming reactions in the actual metabolic networks, while these reactions are reversible and not rate-limiting under ordinal metabolic conditions [16, 18, 20]. Then, multi-step reactions can be reduced to the present form.

The conserved quantities in the CCC model are as follows:

1. The total concentration of the carrier: *c*_pool_ = [*c*]_t_ + [c*]_t_.
2. The sum of the concentration of the active carrier and the secondary metabolite (*c*_sum_ = [*c*]_t_ + [*m*_1_]), because the production of one secondary metabolite should require the consumption of exactly one inactive carrier.
3. The difference between the concentration of the inactive carrier and the secondary metabolite: $c_{\text{diff}}^{*}={\left[c^{*}\right]}_{t}-\left[m_{1}\right]$, similar to *c*_sum_.

Note that the number of independent conserved quantities is two because of $c_{\text{pool}}=c_{\text{sum}}+c_{\text{diff}}^{*}$.

### Appearance of slow relaxation

In metabolic networks, some types of inputs will be in a CCC, e.g., changes in the influx and efflux rates of the first metabolite, changes in the ratio between active and inactive carriers due to the environmental changes, and the new synthesis and degradation of a carrier. Here, we consider the condition in which the total carrier concentration is conserved so that the *c*_pool_ does not change. Therefore, we consider changes in *k*_in_ and the ratio between active and inactive carriers as inputs.

**Fig 2:**
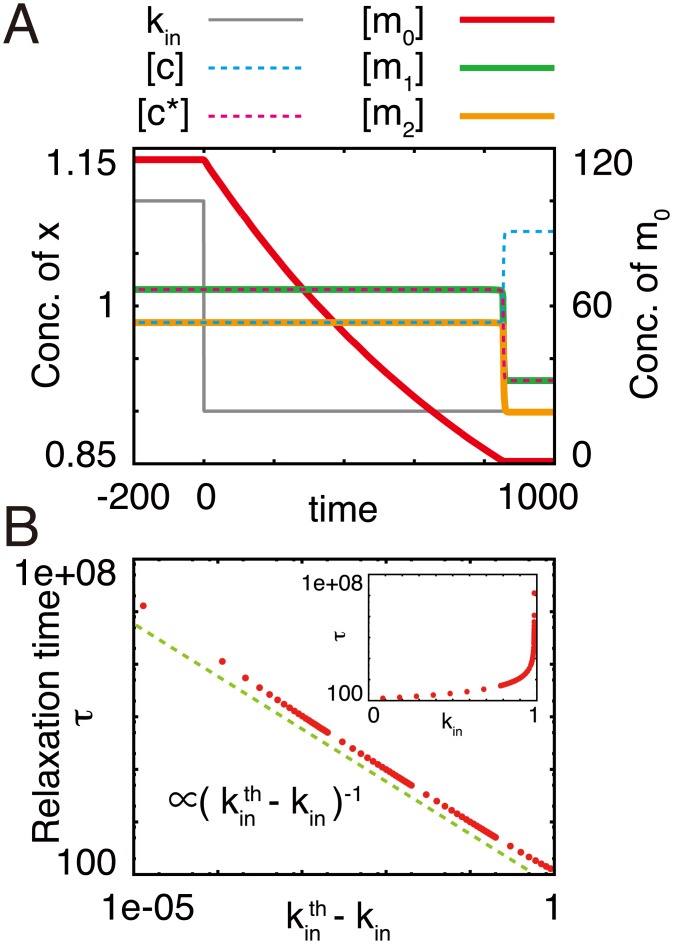
Relaxation of the CCC model after changes in *k*_in_. (A) Time evolution of carriers and the metabolite following the change in *k*_in_. Initially, *k*_in_ was set to 1.1, and the system reached the steady state. Subsequently, *k*_in_ was changed from 1.1 to 0.9 at time = 0.0. (B) Changes in the relaxation time with *k*_in_. The initial conditions were set as [*m*_0_] = 100.0, [*m*_1_] = [*m*_2_] = 0.0, [*c*] = *c*_pool_, and [c*] = 0. *k*_leak_ was set to 0. The green dashed line represents ${\left(k_{\text{in}}^{\text{th}}-k_{\text{in}}\right)}^{-1}$, where $k_{\text{in}}^{\text{th}}$ is 0.984313. Inset: Semi-log plot of *τ* vs. *k*_in_.

Initially, we changed the influx rate. We allowed the system to approach a steady state, and then changed *k*_in_ at time = 0 (Fig 2A). When *k*_in_ is altered, the concentrations of all molecules do not change in the timescales of the active carrier consuming and producing reactions (*k*_c_ and *k*_p_ are set as 1.0). Accordingly, the changing of [*m*_0_] is slower than the timescale of *k*_c_ and *k*_p_, while the concentrations of other components do not change as in the quasi-steady state. When [*m*_0_] decreases to become sufficiently small, the concentrations of the others drastically change with a fast timescale, i.e., the timescale of relaxation in [*m*_0_] is approximately one thousand times that of *k*_c_ and *k*_p_. Consequently, the system reaches the true steady state, i.e., the other components relax into the steady-state values in the timescale of *k*_c_ and *k*_p_.

When we change the ratio between [*c*] and [c*], which is considered the change in *c*_sum_, a similar type of behavior is observed (S1 Fig). Although the concentrations of molecules without *m*_0_ change when the ratio changes, the system can reach the quasi-steady state quickly. Then, [*m*_0_] changes slowly and the other components subsequently change rapidly.

The timescale of the relaxation depends on the input strength. We defined the relaxation time *τ* as the time when the sum of differences of the concentrations of all molecules from the previous time point falls below the threshold (10^−7^). When we change *k*_in_, *τ* diverges at the point where $k_{\text{in}}=k_{\text{in}}^{\text{th}}$ (Fig 2B, inset). The relaxation time is proportional to the inverse of the difference between *k*_in_ and $k_{\text{in}}^{\text{th}}$ (Fig 2B) so that the relaxation time critically slows down around the critical point. Note that although the relaxation time is also critically prolonged in the vicinity of saddle-node bifurcation, the exponent is 1/2 [21] and differs from the obtained result.

When the relaxation time slows down, the relaxation manner of [*m*_0_] becomes linear with time rather than exponential (S2 Fig). This can be observed in both cases where *k*_in_ and *c*_sum_ change in the system (Fig 2A and S1 Fig). If both the active carrier-producing and -consuming reactions are reversible, similar slow dynamics appears, while the switch from the quasi-steady state to the true steady state is smooth for small *k*_out_ values (see S3 Fig and section 2 in S1 Text). This may be due to inhibition of the active carrier-producing reaction by binding of *m*_2_ to c*. Therefore, the slow relaxation is considered to be a general behavior of the CCC in response to different environmental changes.

### Origin of the slow relaxation

To elucidate the process of the slow relaxation, we reduced the number of arguments in our model by using conserved quantities. Although our model had five ODEs even when the association and dissociation reactions are eliminated adiabatically, [*m*_2_] is involved only in the equation for [*m*_2_] and not in other equations. Moreover, two equations can be eliminated because of two independent conserved quantities. Hence, the model can be reduced to the following two ODEs:

$$\begin{array}{ccc} \frac{d[m_{0}]}{dt} & = & k_{\text{in}}-k_{c}\frac{\left[m_{0}\right]\left[c\right]}{K_{0}+\left[c\right]}-k_{\text{leak}}\left[m_{0}\right], \end{array}$$ (2a)

$$\begin{array}{ccc} \frac{d[m_{1}]}{dt} & = & k_{c}\frac{\left[m_{0}\right]\left[c\right]}{K_{0}+\left[c\right]}-k_{p}\frac{\left[m_{1}\right]\left[c^{*}\right]}{K_{1}+\left[c^{*}\right]}, \end{array}$$ (2b)

$$\begin{array}{ccc} \left[c\right] & = & \frac{-\beta +{\left\{\beta^{2}+4K_{0}\left(c_{\text{sum}}-\left[m_{1}\right]\right)\right\}}^{1/2}}{2}, \end{array}$$ (2c)

$$\begin{array}{ccc} \left[c^{*}\right] & = & \frac{-\gamma +{\left\{\gamma^{2}+4K_{1}\left(c_{\text{pool}}-c_{\text{sum}}+\left[m_{1}\right]\right)\right\}}^{1/2}}{2}, \end{array}$$ (2d)

where *β* = [*m*_0_] + *K*_0_ − *c*_sum_ + [*m*_1_] and *γ* = *K*_1_ + *c*_sum_ − *c*_pool_.

**Fig 3:**
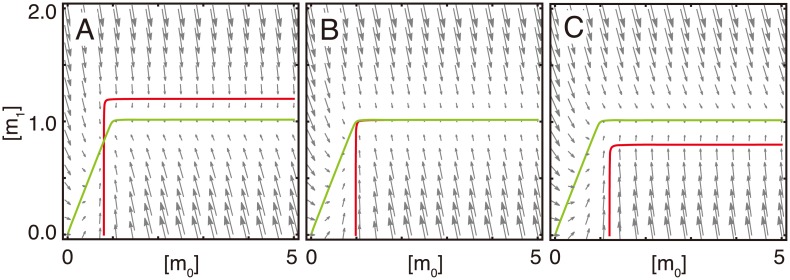
Nullclines of the CCC model. Red and green lines represent the nullclines for [*m*_0_] and [*m*_1_], respectively. Gray arrows are vector fields. *k*_in_ is (A) 0.8, (B) $k_{\text{in}}^{\text{th}}=0.984313$, and (C) 1.2. When *k*_in_ is smaller than $k_{\text{in}}^{\text{th}}$, a stable fixed point exists, whereas there is no stable fixed point and [*m*_0_] will diverge when *k*_in_ is larger than $k_{\text{in}}^{\text{th}}$.

The nullclines for [*m*_0_] and [*m*_1_] are presented in Fig 3, in the case of *k*_leak_ = 0. By altering *k*_in_, the vertical position of the nullcline for [*m*_0_] is changed as well. The CCC behavior changes drastically in the vicinity of the critical point. When *k*_in_ is smaller than $k_{\text{in}}^{\text{th}}$, a stable fixed point appears (Fig 3A), and when *k*_in_ is close to $k_{\text{in}}^{\text{th}}$, relaxation to the fixed point is critically slowed down due to the approach of two nullclines. In this case, a slow manifold is located on the nullcline for [*m*_1_], which is nearly parallel to the nullcline for [*m*_0_]. In the region where two nullclines are parallel, the relaxation speed across the slow manifold is proportional to the distance from the nullcline for [*m*_0_]; i.e., the speed of change in [*m*_0_] is given as a constant that is proportional to $k_{\text{in}}^{\text{th}}-k_{\text{in}}$. Hence, the degradation of *m*_0_ is the rate-limiting process when the distance between the nullclines is sufficiently small, and the relaxation time is proportional to the inverse of $k_{\text{in}}^{\text{th}}-k_{\text{in}}$ (Fig 2B), while the relaxation manner of *m*_0_ depends linearly on time (S2 Fig). However, when *k*_in_ is larger than $k_{\text{in}}^{\text{th}}$, nullclines do not cross anywhere and [*m*_0_] diverges (Fig 3C).

In particular, if *k*_in_ is equal to $k_{\text{in}}^{\text{th}}$, the points on the overlapped nullclines become neutrally stable fixed points (Fig 3B). When *k*_in_ is fixed and *c*_sum_ is changed, both of the nullclines move. Moreover, in this case, the nullclines approach each other for the critical *c*_sum_ value (see S4 Fig), and the slow relaxation occurs.

Here, $k_{\text{in}}^{\text{th}}$ can be obtained analytically for *k*_leak_ = 0 (see section 3 in S1 Text).

$$\begin{array}{ccc} k_{\text{in}}^{\text{th}} & = & \frac{k_{c}k_{p}}{2(k_{c}+k_{p})}\{c_{\text{pool}}+c_{\text{sum}}+\alpha \\ & & +\sqrt{{\left(c_{\text{pool}}-c_{\text{sum}}+\alpha \right)}^{2}+4c_{\text{sum}}\alpha }\}. \end{array}$$ (3)

where *α* = *k*_c_ *K*_1_/(*k*_c_ + *k*_p_). For the limit of *K*_1_ → 0, i.e., *m*_1_ can perfectly bind to c* and never dissociate, Eq (3) becomes:

$$\begin{array}{c} k_{\text{in}}^{\text{th}}=\{\begin{array}{cc} \frac{k_{c}k_{p}c_{\text{sum}}}{\left(k_{c}+k_{p}\right)} & (c_{\text{pool}}>c_{\text{sum}}),\\ \frac{k_{c}k_{p}c_{\text{pool}}}{\left(k_{c}+k_{p}\right)} & (c_{\text{pool}}<c_{\text{sum}}). \end{array} \end{array}$$ (4)

Eq (4) represents the maximal capacity of the flux of CCC. Therefore, when the influx exceeds the capacity, the CCC can become jammed, which leads to the appearance of slow dynamics.

If *k*_leak_ is not zero, the nullcline for [*m*_0_] is tilted, and a fixed point is obtained for large *k*_in_ (S5 Fig). For a system with a finite *k*_leak_ value, $k_{\text{in}}^{\text{th}}$ cannot be defined. However, in the parameter range where *k*_in_ is close to the $k_{\text{in}}^{\text{th}}$ for the system with no *k*_leak_, the fixed point value changes drastically and slow relaxation appears, which cannot be represented by bifurcation.

### Robustness and responsiveness to environmental changes

**Fig 4:**
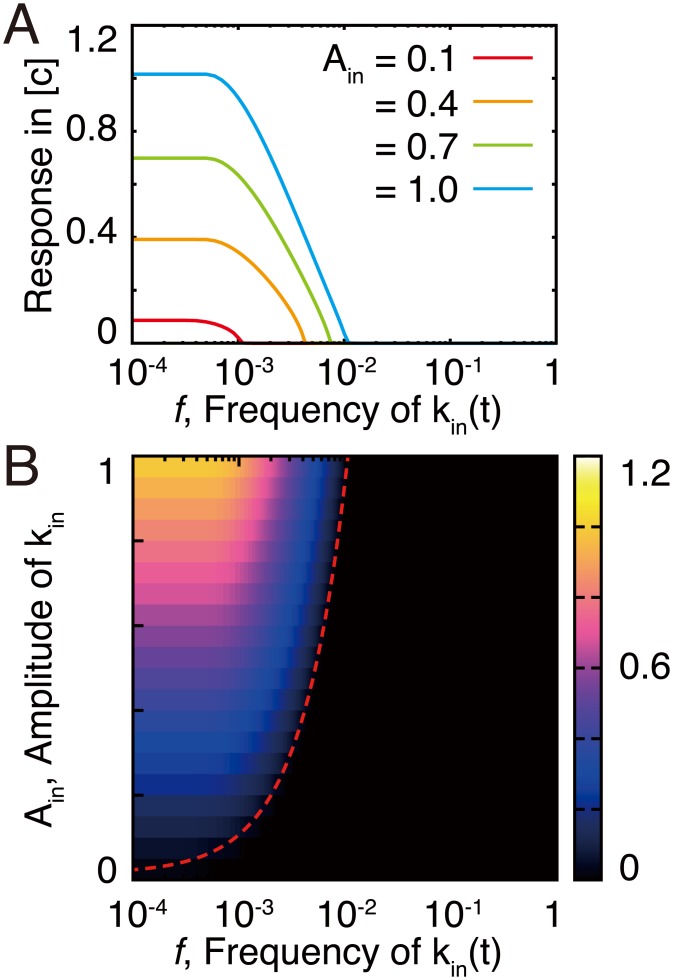
Frequency responses of the CCC model. (A) Changes in the concentration of the active carrier vs. cyclic changes in *k*_in_(*t*). *k*_in_(*t*) is given as $k_{\text{in}}\left(t\right)=A_{\text{in}}\cos\left(2\pi ft\right)+k_{\text{in}}^{0}$, where *A*_in_ and *f* represent the amplitude and the frequency of *k*_in_(*t*), respectively. Here, $k_{\text{in}}^{0}$ equals 1, which is higher than $k_{\text{in}}^{\text{th}}$, and can be calculated in the case of *k*_leak_ = 0, while we set *k*_leak_ = 0.001. We calculated the difference between the maximum and minimum values of [*c*] following the cyclic changes of *k*_in_(*t*). We observed a similar response for [c*], *m*_1_, and *m*_2_. Different colors indicate the responses obtained for different input amplitudes. Magenta, *A*_in_ = 0.1; orange, *A*_in_ = 0.4; green, *A*_in_ = 0.7; blue, *A*_in_ = 1.0. (B) Changes in the concentration of the active carrier with various *A*_in_ and *f* plotted as a color map. The red dotted line indicates the estimated cut-off frequency calculated from Eq (6).

For organisms, robustness against small fluctuations in nutrient uptake is important for maintenance of the intracellular environment. However, in the case of a sudden decrease in the concentration of nutrients, which may lead to starvation, stress-resistant systems should be activated [22, 23]. Here, we analyzed the frequency responses of the CCC using a cyclic nutrient uptake rate *k*_in_(*t*) with different amplitudes (Fig 4). The CCC demonstrates the low-pass filter characteristics with a sharp cut-off frequency for both weak and strong inputs (Fig 4A). For the case when *k*_in_(*t*) is higher than $k_{\text{in}}^{\text{th}}$, *m*_0_ accumulates and the concentrations of the others does not change over time. For the case when *k*_in_(*t*) is lower than $k_{\text{in}}^{\text{th}}$, *m*_0_ decreases slowly, while the concentrations of the other components remains the same. Here, *m*_0_ concentration represents a buffer, and the sharp cut-off frequency can be achieved. The timescale of the decrease depends on the distance between *k*_in_ and $k_{\text{in}}^{\text{th}}$, and thus the filter characteristics depend on the amplitude of inputs (Fig 4B). For inputs with a smaller amplitude, the cut-off frequency becomes lower, but when a larger amplitude of inputs is used, the CCC demonstrates the response against the higher frequency of inputs.

For larger concentrations of *m*_0_, the rates of enzymatic reactions, i.e., the active coenzyme-consuming and -producing reactions, are considered to be constant due to saturation. Therefore, the nullcline for [*m*_1_] can be considered to be nearly constant, while that for [*m*_0_] can be regarded as a linear equation of [*m*_0_] due to a leak term (S5 Fig). When two nullclines are close, the flow rate along the nullclines can be approximated as the distance between the two nullclines, which is slower than the flow rate approaching the nullclines. Accordingly, the two-dimensional dynamics can be reduced into one-dimensional dynamics along the nullcline for [*m*_1_]. We analytically obtained the frequency response of the dynamics. When *k*_leak_ is not as large, the two-dimensional dynamics (Eqs (2a) and (2b)) can be reduced into one-dimensional dynamics of [*m*_0_] (see section 4 in S1 Text).

$$\begin{array}{c} \frac{d[m_{0}]}{dt}=-\frac{k_{\text{leak}}}{k_{c}}\left[m_{0}\right]-\frac{k_{c}c_{\text{sum}}-k_{\text{in}}\left(t\right)}{k_{c}}+\frac{k_{c}c_{\text{sum}}}{\left(k_{c}+k_{p}\right)}. \end{array}$$ (5)

For *k*_in_(*t*) as a sinusoidal $k_{\text{in}}\left(t\right)=A_{\text{in}}\cos\left(2\pi ft\right)+k_{\text{in}}^{0}$, when [*m*_0_] is saturated, the cut-off frequency is given as:

$$\begin{array}{c} 2\pi f=\frac{k_{\text{leak}}k_{c}A_{\text{in}}}{k_{c}k_{\text{in}}^{0}-\left(k_{c}+k_{\text{leak}}\right)k_{\text{in}}^{\text{th}}}-\frac{k_{\text{leak}}}{k_{c}}. \end{array}$$ (6)

The estimated cut-off frequency fits well with the simulation result (red dashed line in Fig 4B), suggesting that the complex dynamics can be reduced to one-dimensional dynamics due to the conserved quantities, and that the necessity for robustness against external fluctuation is determined by the conditions underlying this saturation.

Note that Eq (5) is independent of a form of function of the influx rate. Hence, if the influx rate is regulated by downstream products, the dynamics of the CCC are reduced into one-dimensional dynamics. Although the filter characteristics (Eq (6)) depend on the regulation, robustness by the slow dynamics will always be achieved.

### Robustness against intrinsic noise

**Fig 5:**
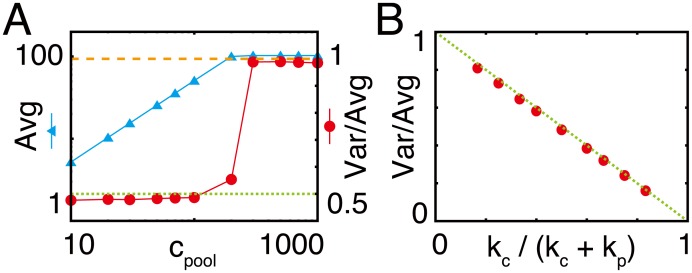
Reduced intrinsic noise in the CCC model. (A) Average and variance per average (Fano factor) of the number of *m*_1_ for different carrier concentrations. The cyan line and triangles represent the average number, and the red line and circles represent the Fano factor. The dashed orange line indicates 1 and the green dotted line indicates 0.5. *k*_in_ is set to 100.0 and *c*_sum_ is the same as *c*_pool_. For sufficiently small values of *c*_pool_, the average number of *m*_1_ decreases linearly and the Fano factor approaches 0.5, while for the large *c*_pool_ the Fano factor is approximately 1. (B) Fano factor of the number of *m*_1_ in various *k*_c_ / (*k*_c_ + *k*_p_). The red circles represent simulation data, and the green dotted line represents an analytical solution. *k*_p_ was fixed to 1, while *k*_c_ was altered to change *k*_c_ / (*k*_c_ + *k*_p_).

To investigate the robustness of the CCC against the intrinsic noise caused by the stochasticity of biochemical reactions, we calculated the stochastic dynamics of the original full model with consideration of complex formation using the Gillespie algorithm [24], which can generate a statistically possible trajectory of the solution of stochastic equations and is often used for modeling biochemical reactions. We calculated the long trajectories of the solution and several statistics from the given trajectories. When the *c*_pool_ is larger than the critical value, the average number of *m*_1_ is almost constant for different *c*_pool_ values. In this condition, the Fano factor, which is the ratio of the variance to the average, is approximately 1, as in similar non-catalytic reactions. However, below the critical point where the flux is limited by the concentration of the carrier, i.e., the first metabolite is saturated, the Fano factor of the number of *m*_1_ decreases below 1 (Fig 5A). This suggests that the carrier cycling can reduce the variance of the concentration of intermediate metabolites.

To investigate the mechanisms underlying the decrease in the intrinsic noise, we analyzed the probabilistic dynamics of the number of *m*_1_ molecules, *n* (see section 5 in S1 Text). Under the not-saturated condition, i.e., when the *m*_0_ concentration is lower than the maximal coenzyme concentration *c*_max_, which is the same as *c*_sum_ when *c*_sum_ < *c*_pool_, only the consumption rate of *m*_0_ is proportional to *n* but the production rate is not. Therefore, the steady-state distribution of the number of *m*_1_ is given as a Poisson distribution [25] in the limit of *K*_0_ → 0 and *K*_1_ → 0; i.e., the metabolites can bind to coenzymes perfectly. Thus, the Fano factor becomes 1, which is similar to the previously reported condition [26].

In the saturated condition, i.e., the *m*_0_ concentration is higher than *c*_max_, the production rate becomes *k*_c_(*c*_max_ − *n*) due to conservation of the coenzyme, while the consumption rate remains the same as above. Here, both the production and consumption rates are proportional to *n*, which is considered the feedback-regulated production through the conserved quantity. The steady-state distribution represents the binomial distribution [25] and the Fano factor is given as:

$$\begin{array}{c} \frac{\sigma^{2}}{<n>}=1-\frac{k_{c}}{k_{c}+k_{p}}. \end{array}$$ (7)

Therefore, the fluctuation is reduced depending on the *k*_c_ and *k*_p_ values (Fig 5B), and the recycling of carriers improves the signal-to-noise ratio by feedback regulation via the moiety conservation. This effect does not depend on the concentration of the coenzyme as long as the metabolite is saturated before the coenzyme consumption step. The feedback can also reduce the fluctuation in the active and inactive carrier concentrations.

Note that when both the production and consumption rates of *m*_1_ obey the Michaelis-Menten reaction with different coenzymes, the number of *m*_0_ shows a random walk in the range of 0 to ∞, or becomes zero, or diverges, depending on the parameter settings. In any case, the Fano factor never falls below 1 (see section 5 in S1 Text).

## Discussion

Here, we proposed a minimal model of metabolic systems that includes recycling of the moiety-conserved carriers with complex formation between carriers and other metabolites. In transient dynamics, the conserved quantities constrain a dimension of orbits moving on to the steady state, similar to the steady-state solution space, and dynamics in the restricted dimension demonstrate various phenomena. These effects can be summarized by two properties: **1) jamming** and **2) feedback**, which are followed by the slow relaxation and robustness against internal and external fluctuations.

We demonstrated that the relaxation dynamics in the CCC are decelerated by the jamming when the nutrient uptake rate is close to the capacity of the cascade. Such slow relaxation to the steady state has also been discussed in other enzymatic networks [17]. From the viewpoint of dynamical systems theory, this jamming is due to a restriction of the phase space and the closure of nullclines by the conservation of carrier concentration. In this situation, the concentrations of internal metabolites, the final product, and active and inactive carriers are almost constant and can be drastically different from the concentrations in the steady state. Hence, if a metabolic system seems to be in a steady state on a short timescale after an environmental change, this system will not always be in the true steady state but rather in a quasi-stable state. Consequently, the previous theoretical studies considering steady metabolic states were not able to analyze the cellular metabolism in these quasi-stable states.

Such jamming in the metabolic flow is likely to be observed in several metabolic systems, including the glycolytic cascades. In fact, some mutants of the budding yeast exhibit growth defects, which were explained due to the abnormal accumulation of intermediate metabolites based on computer simulations [18]. Such time evolution is consistent with our results caused by the jamming, while we further uncovered the origin of this phenomenon using dynamical systems theory. This study suggests the possibility that the native metabolic cascade resides in the vicinity of the critical point and can be easily jammed by genetic perturbations, which will be validated in future studies.

Furthermore, we suggest that the jamming mechanism may represent the mediator between molecular- and organism-level timescales [27]; i.e., the jamming slows down the fast enzymatic turnover and may determine the timescale of physiological behaviors. The slow relaxation can help organisms maintain their cellular condition against changes in the nutrient condition and will work as a memory of past environmental change. For example, the slow timescale might be related to a slow response of the relaxed strain against carbon and amino acid starvation [22, 23].

We have demonstrated the robustness of metabolic systems against external fluctuation, which opens the door for further theoretical studies of the quasi-steady state of metabolic dynamics. The concentrations of some metabolites and carriers are maintained throughout small fluctuations in the influx rates. At the same time, the CCC is responsible for the large alterations in the influx rate. Therefore, the CCC shows both robustness against small fluctuations and responsiveness to large fluctuations; i.e., the CCC does not respond to small and short-term changes in the nutrient uptake rate, but it can rapidly respond to larger alterations in nutrient uptake rates. This robustness and responsiveness are most likely due to the wide range of the quasi-steady states in the phase space and the input-dependent timescales of relaxation. These two properties depend on the non-linear dynamics owing to the complex formation that is not in the vicinity of a fixed point, which has not been investigated using a constraint-based approach or with steady-state analysis of the previous model. A relationship between robustness and responsiveness has also been studied in other systems [28] and will be further investigated in various biological systems.

To measure quasi-steady state characteristics, the dynamics of metabolites should be measured non-invasively for a long time; however, the real-time measurement techniques of metabolites are insufficient at present. Thus, we expect that the continuous development of new measurement techniques will help to validate our predictions experimentally.

We demonstrated that an internal metabolite regulates its production via feedback mechanisms by the moiety conservation. The noise in the concentrations of metabolites and carrier can be reduced drastically. This feedback mechanism is important for stabilization of the metabolite and carrier concentrations at the finite constant values during the slow relaxation. When this feedback is missing, this noise cannot be reduced microscopically. Therefore, the concentration of the internal molecule should decrease to zero or diverge in the saturated condition (S6 Fig and section 5 in S1 Text). This suggests that the feedback mechanism involving the carrier cycling may be responsible for the existence of the quasi-steady states.

There are several important parameters contributing to the robustness against both external and internal fluctuations. In particular, *k*_c_, the turnover rate of an enzyme in the active carrier-consuming reaction, and *k*_p_, that in the active carrier-producing reaction, are important. We estimated these turnover rates using data from previously published studies: the glycolytic pathway of *Escherichia coli* during continuous aerobic cultivation [3, 29] and the lactic acid fermentation pathway of *Lactococcus lactis* during anaerobic cultivation with an 80% lactic acid yield [30–32]. In both pathways, the enzyme levels necessary for each reaction are considered to be sufficient and not a rate-limiting factor, which we assumed in our model, and allowed us to consider the kinetics of substrate and carrier alterations subsequently. Based on our estimation, *k*_*c*_ and *k*_*p*_ in the glycolytic pathway are given as 50 s^−1^ and 12 s^−1^, respectively, while the Fano factor of the internal metabolite is estimated at nearly 0.2. For the lactic acid fermentation pathway, *k*_*c*_ and *k*_*p*_ are given as 1.2 s^−1^ and 4.9 s^−1^, respectively, and the Fano factor is estimated at 0.85 (see Table 1).

**Table 1:** Parameters estimated in metabolic networks.

	Glycolysis of *E. coli*	Fermentation of *L. lactis*
Active carrier production		
Conc. of enzyme	3.21 × 10^−2^ mM [29]	6.0 mM [30]
Conc. of carrier	8.28 × 10^−1^ mM [29]	8.4 mM [30]
Dissociation constant	0.16 mM [3]	0.2 mM [31]
Uptake rate	1.8 mM s^−1^ [29]	
Flux	1.44 mM s^−1^	5.0 mM s^−1^ [30]
*k*_c_	50 s^−1^	0.85 s^−1^
Active carrier consumption		
Conc. of enzyme	1.49 × 10^−1^ mM [29]	1.0 mM [30]
Conc. of carrier	9.65 × 10^−1^ [29]	0.7 mM [30]
Dissociation constant	0.26 mM [3]	0.08 mM [32]
Flux	1.44 mM s^−1^	4.4 mM s^−1^ [30]
*k*_p_	12 s^−1^	4.9 s^−1^

Our estimations are too simplified to allow for a quantitative discussion about the cellular metabolic process, because actual metabolic processes are more complicated than represented by our model. However, the properties observed with our model are preserved even if the details of the model change. Indeed, if two CCCs are coupled through a common carrier pool, the slow relaxation to the steady state is observed (see S7 and S8 Figs, and section 6 in S1 Text). This suggests that the moiety conservation pool can underlie the jamming and the feedback processes that determine the dynamics of more complicated metabolic networks than the CCC. If there are some branches in the CCC, our results would still be qualitatively reproduced, although some quantities would no longer be conserved but rather given as quasi-steady-state values. It has been reported that such branches will change the steady-state characteristics [11], e.g., the bistability and hysteresis, and the slow dynamics might appear in transient dynamics approaching each stable fixed point. We expect that further non-trivial dynamic phenomena will be observed in more complicated metabolic networks with moiety conservation via the jamming and feedback. To investigate the effects of conserved carriers in more complicated metabolic networks quantitatively, both theoretical and experimental investigations are required in the future.

## Models and methods

### Parameter estimation in actual metabolic systems

Our analyses suggest that enzyme turnover rates in metabolic pathways are the essential contributors to the robustness of metabolic dynamics. We estimated the parameters described in actual metabolic pathways, i.e., the glycolytic pathway of *Escherichia coli* during continuous aerobic cultivation [29] and the lactic acid fermentation pathway of *Lactococcus lactis* during anaerobic cultivation with an 80% lactic acid yield [30], and calculated the effects of carrier cycling. We approximated the kinetics of each reaction using the Michaelis-Menten equation. Hence, the concentrations of a substrate and carrier, the dissociation constant, and the speed of flux are necessary for the estimation of *k*_c_ and *k*_p_.

For *k*_c_ of phosphofructokinase (PKF) in the glycolytic pathway, the concentrations of fructose 6-phosphate (F6P) as a substrate and ATP as a carrier used are 3.21 × 10^−2^ mM and 8.28 × 10^−1^ mM, respectively [29]. The dissociation constant is given as 0.16 mM [3]. The flux was estimated from the glucose uptake rate, which is 1.8 mM s^−1^ under the same conditions described previously [29]. However, the entire amount of glucose is not catalyzed by PKF, and 20–40% of glucose is considered to be involved in the pentose phosphorylation pathway [29]. Here, we considered this leak to be 20%, and the flux is estimated as 1.8 × 0.8 = 1.44 mM s^−1^. Subsequently, *k*_c_ is estimated to be approximately 50 s^−1^, using the Michaelis-Menten form. For *k*_p_ of pyruvate kinase (PK), the concentrations of phosphoenolpylvate (PEP) and ADP are 1.49 × 10^−1^ mM and 9.65 × 10^−1^ mM, respectively [29]. The dissociation constant is 0.26 mM [3]. We assumed that the flux of PK is similar to that of PKF, and *k*_p_ is estimated to be approximately 12 s^−1^. From the estimated *k*_c_ and *k*_p_, *σ*^2^/<*n*> of the internal metabolite is determined to be 0.2.

In the same manner, *k*_c_ and *k*_p_ in the lactic acid fermentation pathway can be estimated. For *k*_c_ of glyceraldehyde phosphate dehydrogenase (GAPDH), the concentrations of glyceraldehyde 3-phosphate (GAP) and NAD^+^ are 6.0 mM and 8.4 mM, respectively [30]. The dissociation constant is 0.2 mM [31] and the flux is 5 mM s^−1^ [30]. Therefore, *k*_c_ is estimated to be 0.85 s^−1^. For *k*_p_ of lactate dehydrogenase (LDH), the concentration of pyruvate and NADH are estimated as 1 mM and 0.7 mM, respectively [30]. The dissociation constant is 0.08 mM [32], while the flux is given as 4.4 mM s^−1^ [30]. Therefore, *k*_p_ is estimated to be 4.9 s^−1^, and the *σ*^2^/<*n*> of the internal metabolite is determined to be 0.85.

## Supporting information

S1 Text Models, parameters, and analytical calculations. (PDF)

S1 Fig Relaxation of the CCC model following the changes in *c*_sum_. Initially, *k*_in_ was set to 0.6, and both [*c*] and [c*] were set to 0.5*c*_pool_. Afterward, we let the system achieve the steady state, and [*c*] and [c*] changed to 0.99*c*_pool_ and 0.01*c*_pool_ at time = 0, respectively.
(EPS)

S2 Fig Time evolution of [*m*_0_]. *k*_in_ was set to 0.97. The cyan dashed line is linear with time and the magenta dotted line is exponential with time. The time evolution of [*m*_0_] can be fitted well by a linear line. Inset: Semi-log plot of the time evolution of [*m*_0_].
(EPS)

S3 Fig Relaxation of the reversible model after the changes in *k*_in_. Time evolution of carriers and the metabolite following the change in *k*_in_. Initially, *k*_in_ was set to 1.1, and the system reached a steady state. Afterward, *k*_in_ was changed from 1.1 to 0.9 at time = 0.0. *k*_out_ values were set differently in each case: (A) 1.0, (B) 10.0, and (C) 100.0.
(EPS)

S4 Fig Nullclines of the CCC model with different *c*_sum_ values. Red and green lines represent the nullclines for [*m*_0_] and [*m*_1_], respectively. Gray arrows are vector fields. *c*_sum_ is (A) 2.5, (B) 1.8045, and (C) 1.5.
(EPS)

S5 FigNullclines of the CCC model with a leak of *m*_0_.Red and green lines represent the nullclines for [*m*_0_] and [*m*_1_], respectively. Gray arrows are vector fields. *k*_in_ is (A) 0.8, (B) $k_{\text{in}}^{\text{th}}=0.984313$, and (C) 1.2. *k*_leak_ is set to 0.05.(EPS)

S6 Fig Random walk in the double Michaelis-Menten model. Different colored lines indicate different samples with the same parameter set. Both *k*_c_ and *k*_p_ were set to 1, and both *c*_1_ and *c*_2_ were set to 100.
(EPS)

S7 Fig A scheme of the CCCC for *N* = 2. The model consists of two metabolite and carrier cascades.
(EPS)

S8 FigRelaxation of the CCCC for *N* = 2 following the changes in $k_{\text{in}}^{1}$.(A) Time evolution of the carriers and metabolite following a change in $k_{\text{in}}^{1}$ of the model with a large influx rate for cascade 2 ($k_{\text{in}}^{2}=1$) and a large dissociation constant between *c* and $m_{0}^{2}$ ($K_{0}^{2}=10^{2}$). Initially, $k_{\text{in}}^{1}$ was set to 1.1, and we let the system reach the steady state. Following this, we changed *k*_in_ from 1.1 to 0.9 at time = 0.0. (B) Time evolution of the carriers and metabolite following the change in $k_{\text{in}}^{1}$ of the model with a small influx rate and a large leak rate for cascade 2 ($k_{\text{in}}^{2}=0.1$, $k_{\text{leak}}^{2}=1.0$) and a small dissociation constant between *c* and $m_{0}^{2}$ ($K_{0}^{2}=K_{0}^{1}=10^{-3}$). The graph was obtained following the same procedure as applied in S5A Fig.(EPS)

S1 Code The complete source code of the program, which can be compiled by a standard C compiler without any special libraries. (C)
