# Supplementary material for: Metabolic dynamics restricted by conserved carriers: Jamming and feedback
Source: PLoS Comput Biol. 2017 Nov 7;13(11):e1005847. doi: 10.1371/journal.pcbi.1005847 (PMC5693451; doi:10.1371/journal.pcbi.1005847)
Supplement: S1 Text — (PDF) [file pcbi.1005847.s001.pdf]

Supporting Information

Metabolic dynamics restricted by conserved carriers: jamming and feedback

Tetsuhiro S. Hatakeyama, Chikara Furusawa

1 Original full model

Ordinary differential equations (ODEs) of the simplest model of the carrier cycling cascade (CCC) are given as equations of mass action kinetics, as follows:

$$
\begin{aligned}
\frac{d[m_0]}{dt} &= k_{\mathrm{in}} - k_{\mathrm{b0}}[m_0][c] + k_{\mathrm{d0}}[cm_0] - k_{\mathrm{leak}}[m_0], && \text{(1a)}\\
\frac{d[cm_0]}{dt} &= k_{\mathrm{b0}}[m_0][c] - k_{\mathrm{d0}}[cm_0] - k_{\mathrm{c}}[cm_0], && \text{(1b)}\\
\frac{d[m_1]}{dt} &= k_{\mathrm{c}}[cm_0] - k_{\mathrm{b1}}[m_1][c^*] + k_{\mathrm{d1}}[c^*m_0], && \text{(1c)}\\
\frac{d[c^*m_1]}{dt} &= k_{\mathrm{b1}}[m_1][c^*] - k_{\mathrm{d1}}[c^*m_0] - k_{\mathrm{p}}[c^*m_0], && \text{(1d)}\\
\frac{d[m_2]}{dt} &= k_{\mathrm{p}}[c^*m_0] - k_{\mathrm{out}}[m_2], && \text{(1e)}\\
\frac{d[c]}{dt} &= -k_{\mathrm{b0}}[m_0][c]_f + k_{\mathrm{d0}}[cm_0] + k_{\mathrm{p}}[c^*m_0], && \text{(1f)}\\
\frac{d[c^*]}{dt} &= -k_{\mathrm{b1}}[m_1][c^*]_f + k_{\mathrm{d1}}[c^*m_0] + k_{\mathrm{c}}[cm_0], && \text{(1g)}
\end{aligned}
$$

where m_i represents the i-th metabolite, c and c^* are the active and inactive carriers, respectively, and $[x]$ denotes the concentration of x. m_0 and m_1 can form complexes with the active and inactive carriers as cm_0 and c^*m_1 with the association rates k_{b0} and k_{b1}, respectively, and these complexes can decompose with the dissociation rates k_{d0} and k_{d1}, respectively. Active carriers are consumed with the rate k_{c} when m_1 is transformed from m_0 and are produced with rate k_{p} when m_2 is transformed from m_1. m_0 is supplied and diluted with rates k_{in} and k_{leak}, respectively, and m_2 is diluted with rate k_{out}. If we assume that $k_{\mathrm{c}}, k_{\mathrm{p}} \ll k_{\mathrm{b}i}, k_{\mathrm{d}i}$, these equations can be reduced to five ODEs, and we calculated the five-variable model.

Here, we use the parameters presented in S1 Table unless otherwise noted.

TableS. 1: Parameters used in the simple CCC model.

Parameter	Value
c_{pool}	2.0
c_{sum}	2.0
k_{in}	1.0
k_{c}	1.0
k_{p}	1.0
k_{leak}	0.001
K_0	10^{-3}
K_1	10^{-3}

2 Reversible model

ODEs of the reversible model are given as follows:

$$\frac{d[m_0]}{dt} = k_{\text{in}} - k_{\text{c}}\frac{[m_0][c]}{K_0+[c]} + k_{\text{r1}}\frac{[m_1][c^*]}{K_{\text{r1}}+[c^*]} - k_{\text{leak}}[m_0], \tag{2a}$$

$$\frac{d[m_1]}{dt} = k_{\text{c}}\frac{[m_0][c]}{K_0+[c]} - k_{\text{r1}}\frac{[m_1][c^*]}{K_{\text{r1}}+[c^*]} - k_{\text{p}}\frac{[m_1][c^*]}{K_1+[c^*]} + k_{\text{r2}}\frac{[m_2][c]}{K_{\text{r2}}+[c]}, \tag{2b}$$

$$\frac{d[m_2]}{dt} = k_{\text{p}}\frac{[m_1][c^*]}{K_1+[c^*]} - k_{\text{r2}}\frac{[m_2][c]}{K_{\text{r2}}+[c]} - k_{\text{out}}[m_2], \tag{2c}$$

$$\frac{d[c]_{\text{t}}}{dt} = -k_{\text{c}}\frac{[m_0][c]}{K_0+[c]} + k_{\text{r1}}\frac{[m_1][c^*]}{K_{\text{r1}}+[c^*]} + k_{\text{p}}\frac{[m_1][c^*]}{K_1+[c^*]} - k_{\text{r2}}\frac{[m_2][c]}{K_{\text{r2}}+[c]}, \tag{2d}$$

$$\frac{d[c^*]_{\text{t}}}{dt} = k_{\text{c}}\frac{[m_0][c]}{K_0+[c]} - k_{\text{r1}}\frac{[m_1][c^*]}{K_{\text{r1}}+[c^*]} - k_{\text{p}}\frac{[m_1][c^*]}{K_1+[c^*]} + k_{\text{r2}}\frac{[m_2][c]}{K_{\text{r2}}+[c]}, \tag{2e}$$

$$[c]_{\text{t}} = [c] + \frac{[m_0][c]}{K_0+[c]} + \frac{[m_2][c]}{K_{\text{r2}}+[c]}, \tag{2f}$$

$$[c^*]_{\text{t}} = [c^*] + \frac{[m_1][c^*]}{K_1+[c^*]} + \frac{[m_1][c^*]}{K_{\text{r1}}+[c^*]}, \tag{2g}$$

where k_{r1} and k_{r2} are the speed of reactions from m_1 to m_0 and from m_2 to m_1, respectively. K_{r1} and K_{r2} are the dissociation constants between m_1 and c^* and between m_2 and c, respectively. Here, we use the parameters presented in S2 Table.

TableS. 2: Parameters used in the reversible model.

Parameter	Value
c_{pool}	2.0
c_{sum}	2.0
k_{c}	1.0
k_{p}	1.0
k_{r1}	0.1
k_{r2}	0.1
k_{leak}	0.001
K_0	10^{-3}
K_1	10^{-3}
K_{r1}	10^{-3}
K_{r2}	10^{-3}

3 Analytical calculation of $k_{\mathrm{in}}^{\mathrm{th}}$

The reduced CCC model is given as:

$$\frac{d[m_0]}{dt} = k_{\mathrm{in}} - k_{\mathrm{c}}\frac{[m_0][c]}{K_0+[c]} - k_{\mathrm{leak}}[m_0], \tag{3a}$$

$$\frac{d[m_1]}{dt} = k_{\mathrm{c}}\frac{[m_0][c]}{K_0+[c]} - k_{\mathrm{p}}\frac{[m_1][c^*]}{K_1+[c^*]}, \tag{3b}$$

$$[c] = \frac{-([m_0]+K_0-c_{\mathrm{sum}}+[m_1])}{2} + \frac{\sqrt{([m_0]+K_0-c_{\mathrm{sum}}+[m_1])^2+4K_0(c_{\mathrm{sum}}-[m_1])}}{2}, \tag{3c}$$

$$[c^*] = \frac{-(K_1+c_{\mathrm{sum}}-c_{\mathrm{pool}})}{2} + \frac{\sqrt{(K_1+c_{\mathrm{sum}}-c_{\mathrm{pool}})^2+4K_1(c_{\mathrm{pool}}-c_{\mathrm{sum}}+[m_1])}}{2}. \tag{3d}$$

Here, we obtained $k_{\mathrm{in}}^{\mathrm{th}}$ analytically. When k_{leak} is zero, a nullcline for $[m_0]$ is analytically calculated as:

$$[m_1] = c_{\mathrm{sum}} - \frac{k_{\mathrm{in}}}{k_{\mathrm{c}}} + \frac{K_0 k_{\mathrm{in}}}{k_{\mathrm{in}} - k_{\mathrm{c}}[m_0]}. \tag{4}$$

This nullcline converges into $[m_1] = c_{\mathrm{sum}} - k_{\mathrm{in}}/k_{\mathrm{c}}$ for $[m_0] \to \infty$. Although a nullcline for $[m_1]$ becomes too complicated, analysis of the limit of $[m_0] \to \infty$ is helpful. In these limits, the first term of the right side in Eq.(3b) becomes $k_{\mathrm{c}}(c_{\mathrm{sum}} - [m_1]) = k_{\mathrm{c}}[c]_{\mathrm{t}}$, because the maximum speed can be obtained as a multiplication of the turnover rate of the enzyme and the active carrier concentration. Thus, the nullcline in the limit of $[m_0] \to \infty$ is obtained as:

$$\begin{aligned}[m_1] &= \frac{1}{2(k_{\mathrm{c}} + k_{\mathrm{p}})}\{c_{\mathrm{sum}}(2k_{\mathrm{c}} + k_{\mathrm{p}}) - c_{\mathrm{pool}}k_{\mathrm{p}} - k_{\mathrm{p}}\alpha \\ &\quad + k_{\mathrm{p}}\sqrt{(c_{\mathrm{pool}} - c_{\mathrm{sum}} + \alpha)^2 + 4c_{\mathrm{sum}}\alpha}\}, \qquad (5)\end{aligned}$$

where $\alpha = k_{\mathrm{c}}K_1/(k_{\mathrm{c}} + k_{\mathrm{p}})$. Therefore, from Eqs.(4) and (5), $k_{\mathrm{in}}^{\mathrm{th}}$ is obtained as:

$$\begin{aligned}k_{\mathrm{in}}^{\mathrm{th}} &= \frac{k_{\mathrm{c}}k_{\mathrm{p}}}{2(k_{\mathrm{c}} + k_{\mathrm{p}})}\{c_{\mathrm{pool}} + c_{\mathrm{sum}} + \alpha \\ &\quad + \sqrt{(c_{\mathrm{pool}} - c_{\mathrm{sum}} + \alpha)^2 + 4c_{\mathrm{sum}}\alpha}\}. \qquad (6)\end{aligned}$$

For the limit of $K_1 \to 0$, i.e., in the case where m_1 can perfectly bind to c^* and never unbind, Eq.(6) becomes:

$$k_{\mathrm{in}}^{\mathrm{th}} = \begin{cases} \frac{k_{\mathrm{c}}k_{\mathrm{p}}c_{\mathrm{sum}}}{(k_{\mathrm{c}}+k_{\mathrm{p}})} & (c_{\mathrm{pool}} > c_{\mathrm{sum}}), \\ \frac{k_{\mathrm{c}}k_{\mathrm{p}}c_{\mathrm{pool}}}{(k_{\mathrm{c}}+k_{\mathrm{p}})} & (c_{\mathrm{pool}} < c_{\mathrm{sum}}). \end{cases} \qquad (7a)$$

4 Analytical calculation of the frequency response of the CCC model

To obtain the frequency response analytically, we calculated the nullclines using the large $[m_0]$ limit. Using the limit of $[m_0] \to \infty$, the speed of the active coenzyme-consuming reaction becomes $k_{\mathrm{c}}(c_{\mathrm{sum}} - [m_1]) = k_{\mathrm{c}}[c]_{\mathrm{t}}$. Therefore, the ODE and the nullcline for $[m_0]$ can be approximated for large $[m_0]$ as

$$\frac{d[m_0]}{dt} \simeq k_{\mathrm{in}} - k_{\mathrm{c}}(c_{\mathrm{sum}} - [m_1]) - k_{\mathrm{leak}}[m_0], \qquad (8)$$

$$[m_1] \simeq \frac{k_{\mathrm{leak}}}{k_{\mathrm{c}}}[m_0] - \frac{k_{\mathrm{c}}c_{\mathrm{sum}} - k_{\mathrm{in}}}{k_{\mathrm{c}}}, \qquad (9)$$

respectively. A nullcline for $[m_1]$ in the large $[m_0]$ and small K_1 limit is calculated from Eq.(5) as

$$[m_1] \simeq \frac{k_c c_{\mathrm{sum}}}{k_c + k_p} = \frac{k_{\mathrm{in}}^{\mathrm{th}}}{k_p}. \tag{10}$$

Therefore, from Eqs.(9) and (10), the fixed point value of $[m_0]$ is given as:

$$\begin{aligned} [m_0]^* &= \frac{k_{\mathrm{in}}}{k_{\mathrm{leak}}} - \frac{k_c k_p c_{\mathrm{sum}}}{k_{\mathrm{leak}}(k_c + k_p)} \\ &= \frac{k_{\mathrm{in}} - k_{\mathrm{in}}^{\mathrm{th}}}{k_{\mathrm{leak}}}. \end{aligned} \tag{11}$$

When k_{leak} is small, two nullclines are close to the fixed point, and then the speed of change in $[m_0]$ on the nullcline for $[m_1]$ is much slower than that approaching the nullcline and is proportional to the distance between two nullclines. Following this, the two-dimensional dynamics (Eqs.(3a) and (3b)) can be reduced into one-dimensional dynamics of $[m_0]$ around the fixed point as

$$\frac{d[m_0]}{dt} = -\frac{k_{\mathrm{leak}}}{k_c}[m_0] - \frac{k_c c_{\mathrm{sum}} - k_{\mathrm{in}}(t)}{k_c} + \frac{k_c c_{\mathrm{sum}}}{(k_c + k_p)}. \tag{12}$$

When $k_{\mathrm{in}}(t)$ is given as a sinusoidal function $k_{\mathrm{in}}(t) = A_{\mathrm{in}} \cos(2\pi f t) + k_{\mathrm{in}}^0$, $[m_0](t)$ at the steady state is calculated as

$$[m_0](t) = \frac{A_{\mathrm{in}}[(k_{\mathrm{leak}}/k_c)\cos(2\pi f t) + 2\pi f \sin(2\pi f t)]}{(k_{\mathrm{leak}}/k_c)^2 + (2\pi f)^2} + \frac{k_{\mathrm{in}}^0 - k_{\mathrm{in}}^{\mathrm{th}}}{k_{\mathrm{leak}}}. \tag{13}$$

Here, the above approximations are feasible when $[m_0]$ is higher than $[m_0]^{\mathrm{th}} = k_{\mathrm{in}}^{\mathrm{th}}/k_c$ due to saturation, which is the maximal m_0 concentration processed in the first reaction per unit of time. When $[m_0]$ becomes lower than $[m_0]^{\mathrm{th}}$ due to changes in the influx rate, the fixed point is drastically changed and the concentrations of $[m_1]$, $[c]_t$, and $[c^*]_t$ are altered. Therefore, the cut-off frequency is given as the frequency at which the minimal value of Eq.(13) is the same as $[m_0]^{\mathrm{th}}$, as follows:

$$2\pi f = \frac{k_{\mathrm{leak}} k_c A_{\mathrm{in}}}{k_c k_{\mathrm{in}}^0 - (k_c + k_{\mathrm{leak}}) k_{\mathrm{in}}^{\mathrm{th}}} - \frac{k_{\mathrm{leak}}}{k_c}. \tag{14}$$

The estimated cut-off frequency fits well with the result of our simulation (Fig. 4B in the main text), suggesting that the complex dynamics can be reduced into one-dimensional dynamics due to saturation, and the need for robustness against external fluctuation is determined by the conditions underlying this saturation.

5 Internal fluctuation of the metabolite concentration

We considered the stochastic dynamics of the number of m_1 molecules, n. First, we calculated the steady-state distribution of n in a non-saturated condition, i.e., the m_0 concentration is lower than the maximal coenzyme concentration $c_{\rm max}$. Here, we consider the following conditions: $c_{\rm sum} < c_{\rm pool}$ and $c_{\rm max}$ is the same as $c_{\rm sum}$. Using the limits of $K_0 \to 0$ and $K_1 \to 0$, i.e., the metabolites bind to coenzymes perfectly, the production rate of m_1 becomes $k_{\rm c}[m_0]$, and the consumption rate of m_1 becomes $k_{\rm p}n$ when the number of m_1 molecule is n. Here, only the consumption rate is proportional to n, while the production rate is not, so that the master equation can be given as follows:

$$\begin{aligned} \frac{dp_0}{dt} &= k_{\rm p}p_1 - k_{\rm c}[m_0]p_0, \\ \frac{dp_n}{dt} &= k_{\rm p}(n+1)p_{n+1} + k_{\rm c}[m_0]p_{n-1} - (k_{\rm p}n + k_{\rm c}[m_0])p_n, \end{aligned} \tag{15}$$

where p_n is the probability that the number of m_1 molecule is n. The steady-state distribution is the Poisson distribution:

$$p_n^s = \frac{1}{n!}\left(\frac{k_{\rm c}[m_0]}{k_{\rm p}}\right)^n e^{-\frac{k_{\rm c}[m_0]}{k_{\rm p}}}. \tag{16}$$

Therefore, both $< n >$ and σ^2 are given as $k_{\rm c}[m_0]/k_{\rm p}$ and $\sigma^2/ < n >$ becomes 1, which is similar to a previously reported condition [1].

Under the saturated condition, i.e., the m_0 concentration is higher than $c_{\rm max}$, because of coenzyme conservation, the production rate becomes $k_{\rm c}(c_{\rm max} - n)$ while the consumption rate remains the same as the non-saturated condition. Here, both the production and consumption rates are proportional to n. The master equation is thus given as follows:

$$\begin{aligned} \frac{dp_0}{dt} &= k_{\rm p}p_1 - k_{\rm c}c_{\rm max}p_0, \\ \frac{dp_n}{dt} &= k_{\rm p}(n+1)p_{n+1} + k_{\rm c}(c-n+1)p_{n-1} - \{k_{\rm p}n + k_{\rm c}(c-n)\}\, p_n, \\ \frac{dp_{c_{\rm max}}}{dt} &= k_{\rm c}p_{c_{\rm max}-1} - k_{\rm p}c_{\rm max}p_{c_{\rm max}}. \end{aligned} \tag{17}$$

The steady-state distribution is the binomial distribution:

$$p_n^s = \binom{c_{\rm max}}{n} K^n (1+K)^{-c_{\rm max}}, \tag{18}$$

where K is $k_{\mathrm{c}}/k_{\mathrm{p}}$. The moment-generating function is given as:

$$G(k) = \left(\frac{1 + Ke^{k}}{1 + K} \right)^{c_{\mathrm{max}}}, \tag{19}$$

and then,

$$\begin{aligned} < n > &= G'(0) = \frac{c_{\mathrm{max}} k_{\mathrm{c}}}{k_{\mathrm{c}} + k_{\mathrm{p}}}, & (20) \\ < n^2 > &= G''(0) = \frac{c_{\mathrm{max}} k_{\mathrm{c}}}{k_{\mathrm{c}} + k_{\mathrm{p}}} \left\{ 1 + \frac{(c_{\mathrm{max}} - 1) k_{\mathrm{c}}}{k_{\mathrm{c}} + k_{\mathrm{p}}} \right\}, & (21) \\ \sigma^2 &= \frac{c_{\mathrm{max}} k_{\mathrm{c}}}{k_{\mathrm{c}} + k_{\mathrm{p}}} \left(1 - \frac{k_{\mathrm{c}}}{k_{\mathrm{c}} + k_{\mathrm{p}}} \right), & (22) \\ \frac{\sigma^2}{< n >} &= 1 - \frac{k_{\mathrm{c}}}{k_{\mathrm{c}} + k_{\mathrm{p}}}. & (23) \end{aligned}$$

Therefore, the fluctuation can be reduced depending on the k_{c} and k_{p} values (see Fig. 5B in the main text). The carrier cycling can improve the signal-to-noise ratio by feedback regulation through the conserved concentration of a carrier, and this effect does not depend on the concentration of a coenzyme as long as the metabolite is saturated before the coenzyme-consuming step. This suggests that the feedback in the CCC model can reduce the fluctuations of the active and inactive carrier concentrations because of the conserved quantities between $[c]_{\mathrm{t}}$ and $[m_1]$.

To investigate the differences in the CCC from ordinal Michaelis-Menten type reactions, we considered a double Michaelis-Menten model; i.e., reactions from m_0 to m_1 and m_1 to m_2 are catalyzed by different catalysts. Under the non-saturated condition, the behavior of the double Michaelis-Menten model is similar to that of the CCC model, and the Fano factor is approximately 1. However, under the saturated condition, the concentration of m_1 shows a random walk (Fig. S6) when a similar parameter set as used for the CCC model was used, as demonstrated in Fig. 5A in the main text. As shown for the saturated condition, the Fano factor of the m_1 concentration never decreases in the double Michaelis-Menten model.

Furthermore, in the double Michaelis-Menten model, under the non-saturated condition, the master equation is the same as that of the CCC model, and a steady-state distribution is given as the Poisson distribution.

However, under the saturated condition, the master equation is given as

$$\begin{aligned} \frac{dp_0}{dt} &= k_{\mathrm{p}} c_2 p_1 - k_{\mathrm{c}} c_1 p_0, \\ \frac{dp_n}{dt} &= k_{\mathrm{p}} c_2 p_{n+1} + k_{\mathrm{c}} c_1 p_{n-1} - \{k_{\mathrm{p}} c_2 + k_{\mathrm{c}} c_1\} p_n, \end{aligned} \tag{24}$$

where c_1 and c_2 represent the catalysis for the first and second step reactions, respectively. The steady-state distribution is given as

$$p_n^s = \left(\frac{k_{\mathrm{c}} c_1}{k_{\mathrm{p}} c_2}\right)^n p_0^s. \tag{25}$$

Here, if $k_{\mathrm{c}} c_1 / k_{\mathrm{p}} c_2$ is higher than 1, the time evolution of the number of m_1 is governed by the asymmetric random walk and the number of m_1 will diverge. If $k_{\mathrm{c}} c_1 / k_{\mathrm{p}} c_2$ is 1, the time evolution of the number of m_1 is governed by the symmetric random walk from 0 to ∞, as shown in S6 Fig. If $k_{\mathrm{c}} c_1 / k_{\mathrm{p}} c_2$ is lower than 1, the steady-state distribution can be given as a geometric distribution:

$$p_n^s = \left(1 - \frac{k_{\mathrm{c}} c_1}{k_{\mathrm{p}} c_2}\right) \left(\frac{k_{\mathrm{c}} c_1}{k_{\mathrm{p}} c_2}\right)^n. \tag{26}$$

The average and variance are:

$$< n > = \frac{k_{\mathrm{c}} c_1}{k_{\mathrm{p}} c_2 - k_{\mathrm{c}} c_1}, \tag{27}$$

$$\sigma^2 = \frac{k_{\mathrm{c}} c_1 k_{\mathrm{p}} c_2}{(k_{\mathrm{p}} c_2 - k_{\mathrm{c}} c_1)^2}, \tag{28}$$

$$\frac{\sigma^2}{< n >} = \frac{k_{\mathrm{p}} c_2}{k_{\mathrm{p}} c_2 - k_{\mathrm{c}} c_1} = \frac{1}{1 - k_{\mathrm{c}} c_1 / k_{\mathrm{p}} c_2} > 1. \tag{29}$$

Therefore, in the double Michaelis-Menten model, $\sigma^2 / < n >$ should be higher than 1 in all cases.

6 Coupled carrier cycling cascade (CCCC) model

We considered the condition in which two carrier cycling cascades are coupled through a common coenzyme pool. Association and dissociation reactions

between the coenzyme and substrates are eliminated adiabatically, as in the single cascade model, and the ODEs can be given as:

$$\frac{d[m_0^i]}{dt} = k_{\mathrm{in}}^i - k_{\mathrm{c}}^i \frac{[m_0^i][c]}{K_0^i + [c]} - k_{\mathrm{leak}}^i [m_0], \tag{30a}$$

$$\frac{d[m_1^i]}{dt} = k_{\mathrm{c}}^i \frac{[m_0^i][c]}{K_0^i + [c]} - k_{\mathrm{p}}^i \frac{[m_1^i][c^*]}{K_1^i + [c^*]}, \tag{30b}$$

$$\frac{d[m_2^i]}{dt} = k_{\mathrm{p}}^i \frac{[m_1^i][c^*]}{K_1^i + [c^*]} - k_{\mathrm{out}}^i [m_2^i], \tag{30c}$$

$$\frac{d[c]_{\mathrm{t}}}{dt} = \sum_{i=1}^{N} \left\{ -k_{\mathrm{c}}^i \frac{[m_0^i][c]}{K_0^i + [c]} + k_{\mathrm{p}}^i \frac{[m_1^i][c^*]}{K_1^i + [c^*]} \right\}, \tag{30d}$$

$$\frac{d[c^*]_{\mathrm{t}}}{dt} = \sum_{i=1}^{N} \left\{ k_{\mathrm{c}}^i \frac{[m_0^i][c]}{K_0^i + [c]} - k_{\mathrm{p}}^i \frac{[m_1^i][c^*]}{K_1^i + [c^*]} \right\}, \tag{30e}$$

$$[c]_{\mathrm{t}} = [c] + \sum_{i=1}^{N} \frac{[m_0^i][c]}{K_0^i + [c]}, \tag{30f}$$

$$[c^*]_{\mathrm{t}} = [c] + \sum_{i=1}^{N} \frac{[m_1^i][c^*]}{K_1^i + [c^*]}, \tag{30g}$$

where i represents an index of cascade numbers. Although we set N to 2, i.e., two CCCs are coupled (S7 Fig), the obtained results do not change for higher N when the following conditions are satisfied.

The CCCC shows a qualitatively similar response to the environmental changes as the CCC when one cascade is a major pathway. Here, we considered two examples:
1. c and m_0^2 with a larger dissociation constant than c and m_0^1.
2. The influx rate (leak rate) of m_0^2 is smaller (larger) than that of m_0^1.
In both cases, cascade 1 is the major cascade, and the CCCC displays responses to environmental changes in a similar way as observed for the CCC (S8 Fig). Therefore, our findings should be applicable to more complicated metabolic networks as well.

For these, we used the parameter set presented in S3 Table, unless otherwise specified.

TableS. 3: Parameter set used in a CCCC model

Parameter	Value
c_{pool}	2.0
c_{sum}	2.0
k_{in}^2	1.0
k_{c}^1	1.0
k_{c}^2	1.0
k_{p}^1	1.0
k_{p}^2	1.0
k_{leak}^1	0.001
k_{leak}^2	0.001
K_0^1	10^{-3}
K_0^2	10^{-3}
K_1^1	10^{-3}
K_1^2	10^{-3}

References

[1] Levine E, Hwa T. Stochastic fluctuations in metabolic pathways. Proc Natl Acad Sci USA. 2007; 104: 9224-9229.

7 Supporting Figures

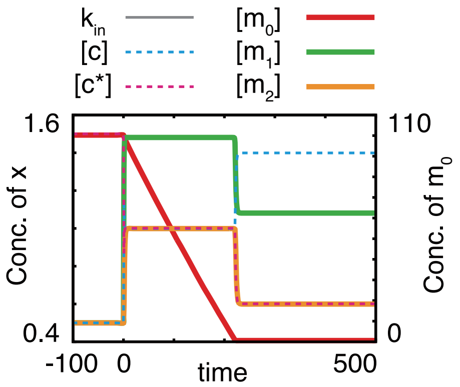

k_in
[c]
[c*]
[m_0]
[m_1]
[m_2]
1.6
0.4
110
0
Conc. of x
Conc. of m_0
-100
0
500
time

FigS. 1

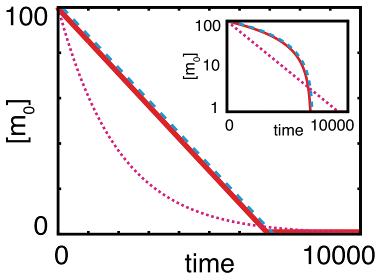

100
0
[m_0]
0
time
10000
100
10
1
[m_0]
0
time
10000

FigS. 2

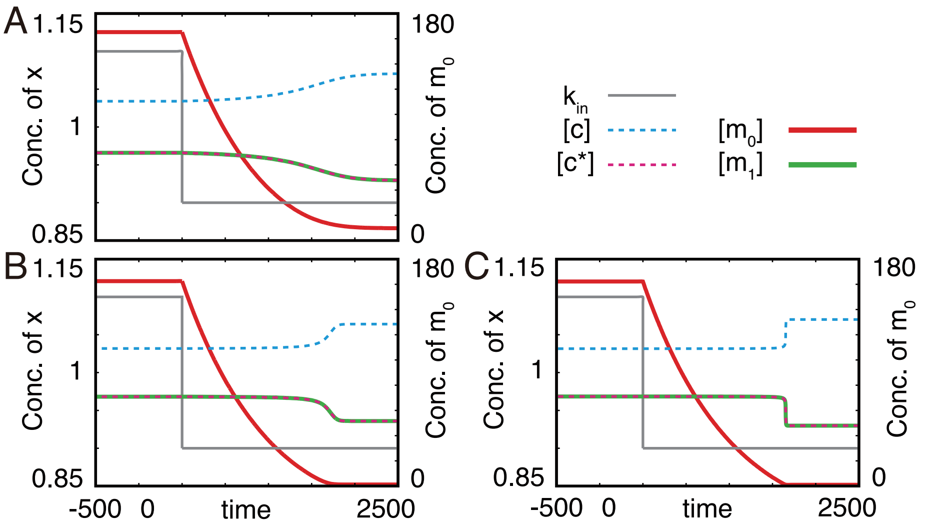
A
B
C
1.15
1
0.85
180
0
Conc. of x
Conc. of m0
-500
0
time
2500
kin
[c]
[c*]
[m0]
[m1]

FigS. 3

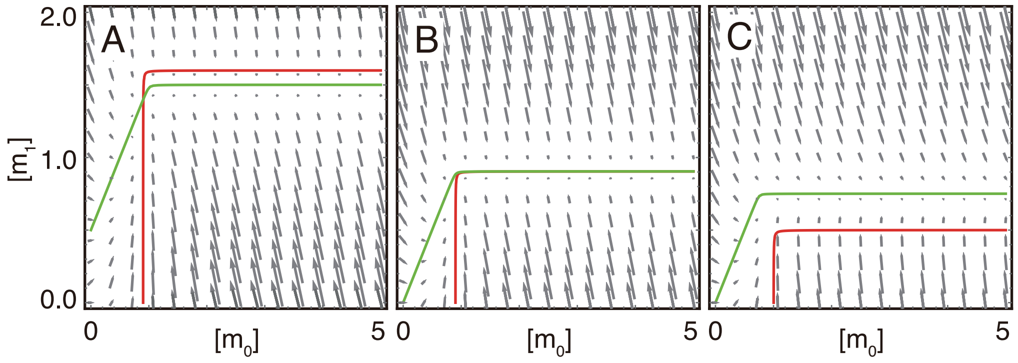
A
B
C
2.0
1.0
0.0
[m1]
0
5
[m0]

FigS. 4

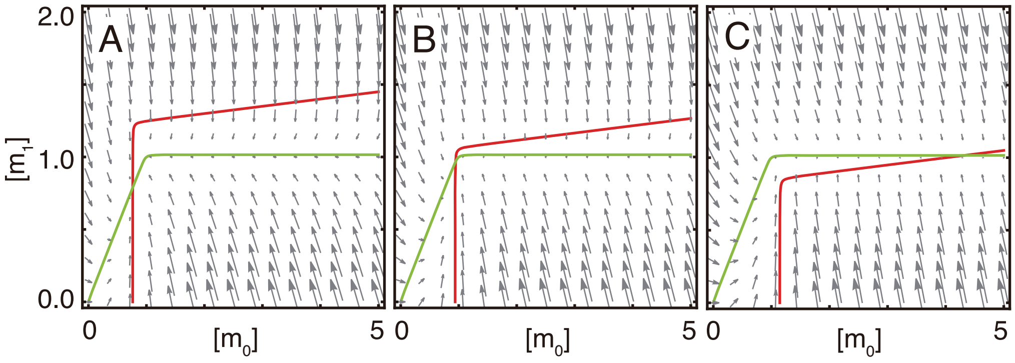

FigS. 5

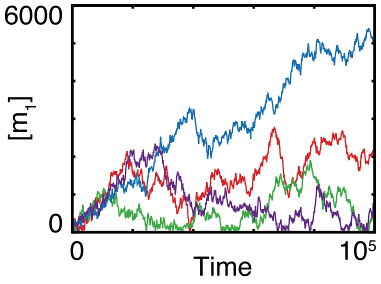

FigS. 6

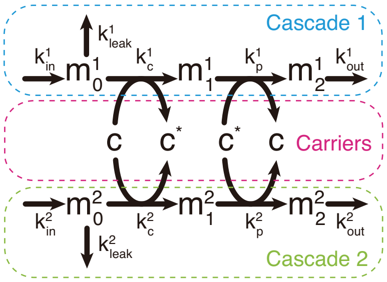

FigS. 7

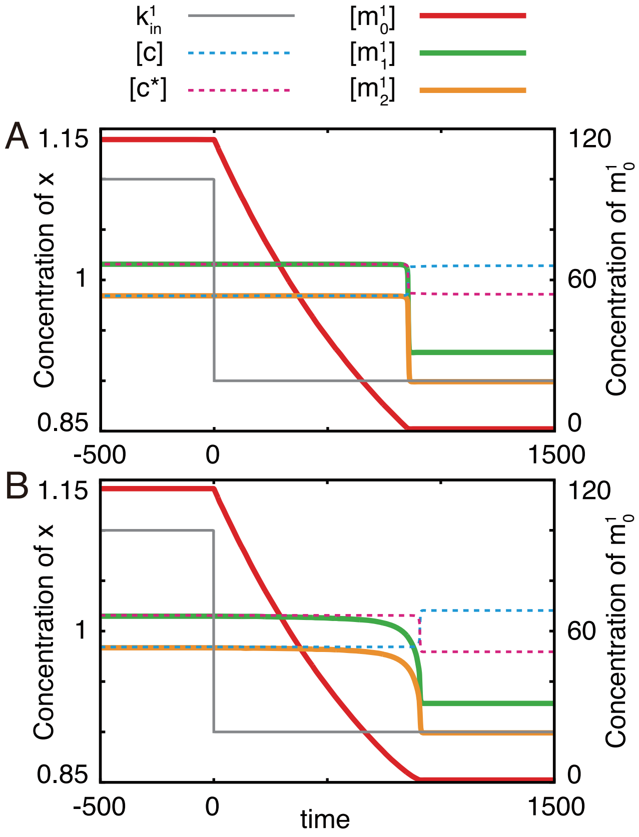

k$^{1}_{in}$
[c]
[c*]
[m$^{1}_{0}$]
[m$^{1}_{1}$]
[m$^{1}_{2}$]
A
B
Concentration of x
Concentration of m$^{1}_{0}$
1.15
1
0.85
120
60
0
-500
0
1500
time

FigS. 8
